# Vaccination Against Respiratory Infections in Adults with Cancer: A Concise Guide for Clinicians

**DOI:** 10.3390/vaccines14010105

**Published:** 2026-01-21

**Authors:** Kay Choong See

**Affiliations:** Division of Respiratory and Critical Care Medicine, Department of Medicine, National University Hospital, Singapore 119228, Singapore; kaychoongsee@nus.edu.sg

**Keywords:** COVID-19 vaccines, immunocompromised host, immunotherapy, influenza vaccines, neoplasms, pneumococcal vaccines, respiratory tract infections, vaccination

## Abstract

Global cancer incidence reached 20 million new cases across 185 countries in 2022, with approximately 10 million cancer-related deaths annually. Among adults with solid tumors and hematological malignancies, infections are a major contributor to morbidity and mortality, with respiratory infections playing a particularly significant role. These infections not only reduce life expectancy but can also delay cancer therapy, negatively affect treatment outcomes, and increase healthcare costs. In recent years, the burden of respiratory infections in this population has been driven by influenza virus, SARS-CoV-2, respiratory syncytial virus, *Streptococcus pneumoniae*, and *Bordetella pertussis*. Effective vaccines are available for all these pathogens and are recommended for adults with cancer, yet vaccination uptake remains suboptimal despite their heightened vulnerability. This review provides practical guidance for healthcare professionals on vaccinating adults with cancer against respiratory infections, summarizing key information to help clinicians address vaccination-related complacency, confidence, and convenience. Evidence from studies in both the general population and cancer patients consistently shows that vaccination benefits outweigh potential risks, with adverse event rates comparable to those seen in individuals without cancer. Early vaccination is encouraged, as there is limited justification for delaying immunization even when immune responses may be reduced. Vaccine dosing aligns with recommendations for the general population, with important exceptions. Live attenuated vaccines should be avoided because of the risk of replication and disease in immunocompromised patients, and selected groups may require booster doses to achieve adequate protection. Notably, cancer immunotherapy does not appear to impair vaccine-induced immune responses.

## 1. Introduction

GLOBOCAN (Global Cancer Observatory) estimates, provided by the International Agency for Research on Cancer, indicate that the incidence of new cancer cases increased to 20 million across 185 countries in 2022 [1], up from 19.3 million in 2020 [2]. Approximately 10 million cancer-related deaths occur each year. According to the Global Burden of Disease Study, cancer incidence and mortality are projected to continue increasing through 2050 [3].

Among adults with solid tumors and hematological malignancies, infections—particularly respiratory infections—significantly contribute to reduced life expectancy [4,5,6,7,8]. In recent years, a number of key pathogens, such as influenza virus, Severe Acute Respiratory Syndrome Coronavirus 2 (SARS-CoV-2, the causative agent of Coronavirus Disease 2019 (COVID-19)), respiratory syncytial virus (RSV), *Streptococcus pneumoniae* (pneumococcus), and *Bordetella pertussis*, have comprised the majority of reported cases [9,10,11,12,13,14]. Additionally, *Haemophilus influenzae* type B is primarily relevant in adult patients undergoing stem cell transplantation for hematologic cancers. While effective therapeutic interventions are available for the principal bacterial infections—namely pneumococcus, *B. pertussis*, and *H. influenzae* type B—antiviral treatment options for the predominant viral pathogens remain limited and necessitate timely administration in cases of severe infection. Although antiviral options are restricted, they play a critical role in influenza and COVID-19 management, and passive antibody therapy is available for RSV; nevertheless, prevention is generally preferable to avoid the risks and side effects associated with treatment, and antiviral agents for influenza can also serve as effective prophylaxis.

Vaccination is presently available to protect against all these pathogens and is advised for all adult cancer patients. However, coverage among this population remains suboptimal, despite their heightened vulnerability to respiratory infections [15,16,17,18,19]. A Danish population-based cohort study published in 2021 found that less than 2% of individuals aged 15 years or older with hematological malignancies received pneumococcal vaccination [20]. For COVID-19 vaccination, patients with cancer also have low uptake of booster doses, despite evidence that booster vaccination is associated with a 39% reduction in all-cause mortality [21]. It should be noted that revaccination entails initiating a complete vaccine series to re-establish immunity, while a booster dose refers to an extra injection administered following the primary series to enhance or extend current protection.

According to the World Health Organization’s Strategic Advisory Group of Experts on Immunization (SAGE) [22], factors such as complacency, doubts about vaccine effectiveness and safety, and logistical challenges may contribute to poor uptake. Clinicians should be aware of these obstacles and actively work to improve immunization rates in adults with cancer. This review provides practical guidance for healthcare professionals on vaccinating adults with cancer against respiratory infections and summarizes key information to help clinicians address vaccination-related complacency, confidence, and convenience.

## 2. Immunological Considerations

Patients are at greater risk of infection if they encounter pathogens and cannot effectively prevent these pathogens from invading. They also face a higher chance of developing severe illness if their immune systems fail to control or eliminate the infection. Increased exposure can result from frequent interactions with healthcare environments and inadequate infection control, both of which are common for cancer patients undergoing monitoring and treatment. Factors that hinder the ability to block pathogen invasion and combat disease include weakened immunity caused by cancer itself and by treatments that inadvertently suppress the immune system [12]. Importantly, frailty, reduced physiological reserve, and impaired recovery associated with cancer and chemotherapy further increase the risk of morbidity and mortality.

Impaired immunity diminishes vaccine efficacy, particularly with increasing levels of immunosuppression, affecting both B-cell and T-cell responses [23]. Individuals undergoing surgery or radiotherapy typically exhibit favorable vaccine responses, with seroconversion rates exceeding 60%, relative to healthy controls. Those receiving chemotherapy or immunotherapy demonstrate intermediate outcomes, with seroconversion rates in the range of 40–60%. In contrast, stem cell transplant recipients requiring immune ablation, as well as patients receiving B-cell depleting therapies such as rituximab, tend to have reduced responses (<40–60% seroconversion compared to healthy controls) [24]. Suboptimal vaccine response is associated with decreased initial protection and accelerated waning below protective thresholds over time. Strategies to address these challenges may include using high-dose formulations [25,26,27,28,29,30] and incorporating adjuvants to boost vaccine efficacy [31,32,33].

Regarding vaccination safety, the primary immunological consideration is whether live attenuated vaccines are appropriate for the patient. Live attenuated vaccines contain organisms capable of replication, which typically elicit a robust and lasting immune response resulting in protective antibody production. However, these vaccines also pose risks, as their capacity for replication can lead to disease if the host’s immune system fails to control them. Consequently, individuals with cancer or compromised immune function may be vulnerable to uncontrolled vaccine strain-induced infection when administered live attenuated vaccines. In such cases, alternative platforms that cannot replicate or cause disease, such as inactivated, protein subunit, or mRNA vaccines, are recommended [12,18]. When alternative vaccine platforms are unavailable, prophylactic antiviral therapies or monoclonal antibodies may be considered, though these options may likewise be unavailable.

Use of cancer immunotherapy does not appear to blunt the immunologic response to vaccination. For example, a meta-analysis comprising ten cohort studies examining influenza vaccination in cancer patients receiving immune checkpoint inhibitor therapy showed generally positive efficacy outcomes [34]. Separately, a review of 19 cohort studies, one cross-sectional study, and seven case reports showed that, compared with patients receiving chemotherapy, those treated with immune checkpoint inhibitors were significantly more likely to achieve seroconversion after COVID-19 vaccination, with rates comparable to individuals without cancer and to patients receiving targeted therapy [35].

Also, vaccination does not appear to interfere with immunotherapy’s effects. The strongest supporting data come from studies on influenza and COVID-19 vaccines, whereas evidence for other vaccines is limited or lacking. A systematic review of 19 studies involving patients with cancer receiving immunotherapy showed that influenza vaccination was not associated with increased immune-related adverse events and conferred high levels of seroprotection; vaccinated patients conversely demonstrated longer progression-free and overall survival [36]. A cohort study of 394 Chinese patients with lung cancer treated with immune checkpoint inhibitors showed that COVID-19 vaccination did not affect progression-free survival [37]. Similarly, an Italian cohort study showed that regular mRNA vaccine boosters did not increase the risk of cancer progression or mortality among patients with advanced lung cancer receiving immune checkpoint inhibitors. Conversely, patients with high PD-L1 expression appeared to have a doubling of overall survival, from 9.7 to 18.6 months [38].

COVID-19 vaccination may even act as an effective immune modulator, enhancing the impact of cancer immunotherapy. Grippin et al. reported that mRNA vaccines do not interfere with treatment and may, in fact, improve the effectiveness of immune checkpoint inhibitors [39]. In both preclinical models and patient analyses, mRNA vaccination increased type I interferon production and activated immune cells, strengthening tumor antigen recognition. Patients who received the vaccine within 100 days of starting checkpoint inhibitor therapy for cancers such as non-small-cell lung cancer and melanoma showed improved anti-tumor responses and higher survival rates.

## 3. Burden of Respiratory Infections in Adults with Cancer

Cancer patients face heightened immunological susceptibility to infections and more severe clinical manifestations, with respiratory illness being a significant concern [40,41,42]. Viral respiratory infections, particularly influenza, further increase the risk of secondary bacterial infections, including *Staphylococcus aureus* arising from normal nasal flora [43]. Each year, millions of new cases of pneumonia and other lower respiratory tract infections are reported [44], with those diagnosed with lung cancer being particularly vulnerable due to airway alterations or blockages that impede effective mucus clearance and predispose them to post-obstructive pneumonia [45,46]. Furthermore, comorbid chronic obstructive airway disease exacerbates the risk of lower respiratory tract infection [47].

Recent research has identified several key vaccine-preventable pathogens contributing to respiratory infections among individuals with cancer, including influenza virus, SARS-CoV-2, RSV, pneumococcus, *B. pertussis*, and *H. influenzae* [9,10,11,12,13,14]. These pathogens are primarily transmitted via respiratory droplets, and in communities with insufficient vaccination coverage, a single infected individual may initiate outbreaks or epidemics.

Immunocompromised patients face a higher risk of severe complications from respiratory infections because their immune response is weak and they respond poorly to antimicrobial therapy. Adverse outcomes may include diffuse pulmonary inflammation, consolidation, and acute respiratory failure. The most critical scenario, acute respiratory distress syndrome (ARDS), often necessitates intensive care, mechanical ventilation, or advanced modalities such as extracorporeal membrane oxygenation (ECMO) [48,49]. Additional complications, such as lung abscess and empyema, may require prolonged antibiotic therapy and surgical intervention. Systemic inflammation associated with infection can induce thrombotic events and multi-organ damage, potentially resulting in myocardial infarction, cerebrovascular accidents, or renal impairment [50,51,52].

A study conducted at the MD Anderson Cancer Center in the United States [53] reported that over one quarter (27.1%) of hospitalized adults with leukemia or recent bone marrow transplant were diagnosed with a respiratory virus, most frequently RSV, rhinovirus, influenza virus, parainfluenza virus, and adenovirus. Among patients infected with these pathogens, the incidence of pneumonia ranged from 58% to 78%, while mortality rates varied from 22% to 44%.

The infection risks posed by specific respiratory pathogens in adult cancer patients have been described. Cooksley et al. examined serious influenza-related pneumonia using the US Nationwide Inpatient Sample [54]. Compared with the general population, who are not at high risk, patients with cancer had three to ten times higher hospitalization rates across different age groups and were ten times more likely to die. A more recent study by Abdel Rahman using the Surveillance, Epidemiology and End Results database reported similar findings among 3,579,199 patients with cancer diagnosed between 1975 and 2016—the standardized mortality rate for influenza or pneumonia-attributed death within the first year after cancer diagnosis was 1.88 times higher than that of the general population [55].

A US administrative database study of more than half a million adults diagnosed with COVID-19 in 2020 showed that patients requiring cancer treatment had 74% higher odds of death, 69% higher odds of intensive care unit admission, and 19% higher odds of hospitalization compared with individuals without cancer [56]. As expected, these risks were even greater among patients with metastatic solid tumors and hematologic malignancies than among those with nonmetastatic solid tumors. Similarly, Hedberg et al. used data from 10 nationwide Swedish registers and found that the 90-day all-cause mortality from 2020 to 2023 among patients with chronic lymphocytic leukemia and COVID-19 was 1.49 to 2.38 times higher than in the general population across different time periods [57].

Ricco et al. conducted a systematic review encompassing 30 studies with a total of 20,067 bone transplant recipients [58]. Among adults, the pooled attack rate of RSV infection was 8.49%, which was higher than that observed in children (4.79%). Case fatality rates were comparable between adults and children (5.99% versus 5.85%, respectively). In addition, among a mixed population of 353 hospitalized older adults with a mean age of 74 years, 23% of whom had a history of or active cancer, the in-hospital mortality rate for RSV infection was 7.4% [59]. These findings underscore that RSV is not solely a pediatric pathogen but is also associated with substantial mortality among immunocompromised patients with cancer.

RSV infection in adults with cancer may be as severe as, or more severe than, influenza or COVID-19 infection. In one study, Shafat et al. conducted a seven-year retrospective cohort study between 2016 and 2022 involving 440 adults with lymphoma or multiple myeloma and lower respiratory tract infections [60]. They reported comparable 90-day all-cause mortality for infections due to RSV and influenza at 6.5% and 6.6%, respectively. Shafat et al. subsequently studied 503 adults with hematologic malignancies hospitalized for respiratory viral infections in 2023 and 2024. Among these patients, 50% had COVID-19, 26.1% had RSV infection, and 22.2% had influenza [61]. The corresponding 30-day all-cause mortality rates were 9.3%, 7.6%, and 3.4%, respectively. In another study, Wee et al. examined 1823 hospitalized adults with cancer in Singapore, of whom 24% had influenza and 12.9% had RSV infection [62]. Compared with influenza, RSV infection was associated with 141% higher odds of intensive care unit or high dependency admission and 142% higher odds of one-month mortality.

A comprehensive Danish study found that individuals with hematological malignancies face up to a 39-fold increased risk of invasive pneumococcal disease—a severe infection affecting sterile sites such as the pleural cavity, bloodstream, or meninges—compared to the general population [20]. Additionally, a meta-analysis encompassing 46 studies published in 2025 reported consistently elevated incidence risk ratios for invasive pneumococcal disease among adults with hematological malignancies, ranging from 11.1 to 34.7, and identified age as only a minor contributing factor [63]. Supporting these findings, a Canadian population-based cohort study conducted between 2000 and 2004 demonstrated markedly higher rates of invasive pneumococcal disease among adults with hematological and solid organ malignancies compared with non-cancer controls [64]. Incidence rates ranged from 143.6 cases per 100,000 person-years in patients with lung cancer to 673.9 cases per 100,000 person-years in those with multiple myeloma, versus 11 cases per 100,000 person-years in individuals without cancer. Similarly, a Dutch population-based cohort study conducted between 2004 and 2016 indicated that the incidence rates of invasive pneumococcal disease were 482 and 79 per 100,000 patient-years for individuals with hematological and solid organ malignancies, respectively, compared to 15 per 100,000 patient-years among controls without malignancy [65].

The burden of severe respiratory infections due to pertussis and *H. influenzae* in adults with cancer is largely extrapolated from general population data [44,66], as cancer-specific epidemiologic studies are lacking. Nevertheless, published case reports describe disease in this population, underscoring the potential clinical impact of these infections despite the absence of robust population-level data. Florax et al. reported a 16-year-old patient with acute B lymphoblastic leukemia who underwent matched related donor bone marrow transplantation and subsequently developed a lower respiratory tract infection; pertussis was confirmed by molecular testing of pharyngeal specimens [67]. Singh et al. reported five patients with cancer who developed invasive *H. influenzae* infection and additionally summarized 17 cases from a literature review; pneumonia with bacteremia was observed in eight cases [68].

Severe respiratory infections can result in long term sequelae, including impaired cardiac and pulmonary function, reduced functional independence, poorer quality of life, and increased mortality. Long COVID, for example, encompasses persistent physical and psychological symptoms following acute infection [69]. These consequences are often more pronounced in patients with cancer, who face higher risks of infection and related complications. Beyond contributing to premature mortality, severe respiratory infections can also delay cancer therapy [18]. Given the substantial costs and complexity of oncologic treatments, preventing infectious complications is therefore critical. Protecting patients with cancer from respiratory infections and minimizing the latter’s impact should be a priority to improve clinical outcomes and support successful cancer treatment.

## 4. Benefits of Vaccination Against Respiratory Infections Among Adults with Cancer

Vaccine immunogenicity, such as seroconversion rates or antibody titers, is often used as a surrogate for protection. However, these measures do not always correlate directly with vaccine efficacy or clinical effectiveness, as a robust immune response in the laboratory does not guarantee reduced infection, disease severity, or transmission in real-world settings.

Assessing vaccine efficacy, defined as the unbiased measurement of protection under rigorously controlled trial conditions, requires randomized clinical trials. Two Cochrane reviews published in 2025 synthesized available evidence for adults with solid organ and hematologic malignancies. The first review, focusing on adults with solid tumors [70], identified one randomized trial evaluating influenza vaccination, which reported no significant differences in mortality or adverse events up to 15 days following surgery. A separate randomized trial assessing the BNT162b2 mRNA COVID-19 vaccine found a vaccine efficacy rate of 94.4% in reducing COVID-19 infection rates for up to six months after the second dose [71]. No randomized trials were uncovered for RSV, pneumococcal, pertussis, or *H. influenzae* type b vaccinations.

The second review examined adults with hematological cancers [72]. In the previously mentioned randomized trial [71], 95 participants diagnosed with lymphoma, leukemia, or myeloma were assessed. In this small subgroup of patients with hematologic malignancies, one case of COVID-19 occurred in both the vaccinated and unvaccinated arms, resulting in no demonstrable vaccine efficacy. Overall, no reduction in COVID-19 incidence was observed at six months after the second dose compared with placebo or unvaccinated groups; however, the very small sample size precluded definitive conclusions. Furthermore, no randomized trials were identified that compared vaccination with non-vaccination for influenza, RSV, pneumococcal, pertussis, or *H. influenzae* type b vaccines.

In the absence of sufficient randomized trial data, vaccine effectiveness may be estimated using cohort studies that compare vaccinated and unvaccinated individuals. While the majority of vaccination benefit evidence derives from studies in the general population [12,66,73], some research specifically examines adults with cancer. Large cohort studies also enable evaluation of multiple clinically relevant endpoints over extended follow-up periods.

For instance, the INVIDIa-2 multicenter study in Italy showed that among 1004 patients with advanced solid tumors receiving immune checkpoint inhibitors, propensity score-matched analyses demonstrated that, after a median follow-up of 20 months, influenza vaccination was associated with an extension of median overall survival from 20.9 to 27.0 months and median progression-free survival from 9.6 to 12.5 months [74]. Separately, Gögenur et al. conducted a register-based nationwide cohort study in Denmark, which showed that postoperative influenza vaccination administered within 30 days after surgery was associated with an 18% reduction in the odds of overall mortality among patients with solid organ cancers [75]. Amdisen et al. conducted another register-based nationwide cohort study in Denmark. Among 53,249 adults with solid tumors receiving chemotherapy and 22,182 adults with hematologic malignancies, influenza vaccination was associated with a 34% reduction in mortality across five influenza seasons [76].

For COVID-19 vaccination, a UK population-based registry study showed that among adults aged 18 years and older with cancer, overall vaccine effectiveness against breakthrough infections was 65.5% compared with 69.8% in non-cancer controls, declining to 47.0% at 3 to 6 months versus 61.4%, respectively [77]. In the two-year COICA observational study of 141 patients with cancer in Italy, COVID-19 vaccination compared with no vaccination was associated with lower rates of computed tomography (CT)-diagnosed pneumonia (0 versus 48.6%), hospitalization (2.0 versus 15.2%), oxygen therapy (0 versus 14.1%), and mortality (0 versus 7.6%) [78]. Additional booster doses of COVID-19 vaccination may restore waning immunity [79,80]. In Spain, Neto et al. examined two large primary care cohorts comprising more than 100,000 vaccinated and unvaccinated patients with cancer and found that primary COVID-19 vaccination reduced the risk of hospitalization by 51.8% and COVID-19-related death by 77.9% [81]. These protective effects increased to 77.9% and 80.2%, respectively, with booster vaccination [81]. Consistent with serological response data, immunotherapy did not appear to diminish the protective effect of COVID-19 vaccination [82].

For pneumococcal vaccination, a Taiwanese population-based matched cohort study evaluated the effects of the 23-valent pneumococcal polysaccharide vaccine (PPSV23) among 1887 adults aged 75 years and older who survived at least five years post-cancer diagnosis [83]. Of these, 377 received the vaccine and 754 matched controls remained unvaccinated. The vaccinated cohort experienced fewer hospitalizations for pneumonia compared to their unvaccinated counterparts (73.66 vs. 117.82 per 1000 person-years), corresponding to a 30.5% reduction in hospitalization risk, though vaccination did not confer an overall survival benefit. Another Taiwanese population-based matched case–control study found an overall survival benefit among 2188 adults with prostate cancer who received PPSV23 compared with 2188 unvaccinated patients (7-year overall survival rate of 47.5% versus 42.3%, *p* < 0.001) [84].

While no randomized trials have compared pneumococcal conjugate vaccination with no vaccination in adults with cancer, Svensson et al. randomized 128 adult patients (median age 69 years old) with chronic lymphocytic leukemia and demonstrated that the 13-valent pneumococcal conjugate vaccine (PCV13) elicited superior immune responses compared with PPSV23 at one and six months after vaccination [85].

## 5. Safety of Vaccination Against Respiratory Infections Among Adults with Cancer

The evaluation of vaccine safety, like benefit assessment, is grounded in evidence from randomized controlled trials and cohort studies. Inactivated and mRNA vaccines are inherently safe in that they cannot cause infection; however, they may still be associated with adverse effects, as observed with mRNA COVID-19 vaccines. The recent Cochrane systematic reviews published in 2025 reported an absence of serious adverse events among adults with solid organ or hematologic malignancies following administration of influenza and COVID-19 vaccines [70,72].

Multiple cohort studies indicate that vaccines exhibit a strong safety profile. A meta-analysis comprising ten cohort studies examining influenza vaccination in cancer patients receiving immune checkpoint inhibitor therapy identified no significant vaccine-related toxicities [34]. Widespread vaccination during the COVID-19 pandemic showed that safety was consistently observed worldwide. A UK cohort study involving 373 adults with solid organ malignancies observed no severe adverse reactions after mRNA or adenovirus vector COVID-19 vaccination [86]. Additionally, a US study involving 284 patients with solid tumors receiving immune checkpoint inhibitors found that COVID-19 vaccination was not associated with an increased incidence of severe immune-related adverse events [87]. Overall, a systematic review of 28 studies demonstrated that COVID-19 vaccination is safe and well tolerated in patients with cancer [88]. For RSV vaccination, a recent cohort study of 46 allogeneic hematopoietic cell transplant recipients found that the adjuvanted vaccine induced modest seroconversion without any serious adverse effects [89].

## 6. Practical Approach and Improving Vaccination Uptake Among Adults with Cancer

Vaccination is most effective when administered at least two weeks before the initiation of immunosuppressive chemotherapy; however, commencement of cancer therapy should not be delayed solely for vaccination purposes. When pre-chemotherapy vaccination is not possible, evidence from randomized trials suggests that administering vaccines within one week after starting chemotherapy yields favorable results (Table 1). In a study conducted by Wunkes et al. on patients with breast and colorectal cancers, influenza vaccination given five days after initiating adjuvant chemotherapy led to higher antibody titers among breast cancer patients at three and 12 weeks post-vaccination compared to those vaccinated 16 days after chemotherapy onset [90].

In another randomized trial by Keam et al., 97 adults with solid organ malignancies were assigned to receive influenza vaccination on day 1 or day 11 of chemotherapy [91]. Seroprotection rates were comparable between the two groups across all strains (H1N1 67% versus 75%, H3N2 77% versus 80%, and influenza B 21% versus 27%). In a separate study involving colorectal cancer patients, similar antibody responses were noted whether PCV13 administration occurred on day of chemotherapy initiation or two weeks before [92]. While certain studies indicate that vaccine efficacy may be enhanced if administered between chemotherapy cycles instead of at the onset of chemotherapy [93], it is essential not to delay immunization unnecessarily, since safeguarding patients against infection during chemotherapy is of paramount importance.

Vaccine dosing generally follows recommendations for the general population, although selected populations may benefit from booster doses of COVID-19 vaccines to achieve adequate protection (Table 2). As demonstrated by the European Research Initiative on Chronic Lymphocytic Leukemia (ERIC), suboptimal humoral and cellular responses to COVID-19 vaccination can be overcome with one to two additional booster doses, resulting in immune response levels comparable to those achieved after natural infection [94].

Sequencing and prioritization are common considerations for both clinicians and patients. These decisions are guided by prevailing risks, such as prioritizing influenza vaccination during periods of high community influenza activity, the feasibility of safe co-administration, and patient willingness to receive multiple vaccines at the same visit. Although cancer specific data on vaccine coadministration are limited, studies in the general population have shown that inactivated influenza, pneumococcal, and COVID-19 vaccines can be safely and effectively co-administered [73,95,96,97,98,99,100], that pneumococcal vaccines can also be given with the Tdap (tetanus toxoid, reduced diphtheria toxoid and acellular pertussis) vaccine [101,102,103], and that RSV vaccines can be administered alongside inactivated influenza and COVID-19 vaccines [104,105,106,107]. During coadministration of vaccines, administering them at separate anatomical sites, such as one injection in the right deltoid and another in the left deltoid, can reduce the risk and severity of local adverse events [12]. When vaccines are administered on separate days, no minimum waiting period is recommended [12].

To address the decline in vaccine efficacy observed in adults with cancer, revaccination may enhance immunological protection (Table 2). In individuals diagnosed with chronic lymphocytic leukemia, administration of PCV13 and PPSV23 followed by a subsequent PCV13 dose five years later resulted in a serological response that was at least twofold greater [108]. Antibody levels following administration of Tdap vaccine gradually wane over approximately 10 years [109] and therefore pertussis vaccination should be administered decennially [66].

In general, adults with hematological malignancies who undergo ablative chemotherapy followed by stem cell transplantation experience profound loss of humoral immunity, irrespective of prior immunization history [13,110,111,112]. Revaccination is therefore required, typically starting about six months after transplantation, and may involve higher vaccine doses or repeated dosing to achieve adequate immune responses [12,13] (Table 2). Available data for COVID-19 and influenza vaccines indicate that patients undergoing chimeric antigen receptor T-cell (CAR T-cell) therapy directed against B-cell antigens have vaccination needs similar to those for stem cell transplantation [111,113,114,115], particularly for recipients of B cell maturation antigen (BCMA)-targeted CAR T-cell therapy compared with those receiving CD19-targeted CAR T-cell therapy [116].

Multiple strategies have been evaluated to improve vaccination uptake. Overall, these approaches aim to address patient complacency by increasing awareness of the risk of severe disease, strengthen confidence in vaccine safety and efficacy, and enhance convenience in accessing vaccination. One review of COVID-19 vaccine uptake highlighted effective physician-level methods, such as framing messaging appropriately, using persuasive communication focused on safety and benefits for both individuals and communities, sharing personal stories, encouraging open conversations, supporting coadministration with annual influenza vaccines, and offering decision aids or visual materials [117]. System-level approaches that have been successful include tailored health messaging and mass media campaigns, making vaccines available onsite, allowing pharmacists to administer vaccines, integrating standard protocols within healthcare settings, offering incentives, and using chatbots for outreach [117].

For pneumococcal vaccination in immunocompromised patients, David et al. applied a system-based strategy: targeted automated alerts for both clinicians and patients via electronic health records improved PCV13 vaccination rates from 11.9% to 52% and PPSV23 rates from 39.4% to 57.1% [118]. These methods are relevant for patients with cancer. In a systematic review of 15 studies covering influenza, COVID-19, and pneumococcal vaccination, strategies to increase vaccine uptake among adult cancer patients were grouped into three main areas—educational materials and campaigns, reminders, and patient counseling—overlapping with both physician- and system-level interventions [16].

Although the focus of this discussion is on respiratory viruses, it is appropriate to note other vaccinations: for example, recombinant zoster vaccine is recommended to prevent recurrence of shingles, while reimmunization with live attenuated vaccines, such as measles, mumps, and rubella, is generally not advised.

## 7. Vaccination Limitations and Future Directions

Current evidence highlights important gaps in our understanding of how best to achieve robust and durable immune protection in adults with cancer. Future research should prioritize strategies that optimize vaccine-induced immunity from the outset, with the goal of achieving more robust protection and improved clinical outcomes. These strategies may include the use of high-dose formulations [25,26,27,28,29,30], combined or sequential administration of multiple standard-dose vaccines [119,120,121], and incorporation of vaccine adjuvants [31,32,33]. Passive antibody therapy, such as monoclonal antibodies for RSV, and antiviral agents, such as oseltamivir for influenza, if available, should also be explored as alternative strategies for individuals at risk of exposure or during the period before protective immune responses develop following immunization. Further studies examining serological responses and the durability of immunity to various vaccines in patients with cancer receiving newer therapies, such as CAR T-cell therapy, are needed to better define initial vaccination and revaccination schedules. Note that RSV vaccination in adults with cancer is supported by limited data specific to this population. Current evidence is still evolving and is largely extrapolated from studies in older adults or the general population.

Beyond vaccination, there is a need to elucidate the role of adjunctive preventive measures. These include nonpharmaceutical interventions such as hand hygiene, while the magnitude and consistency of protection conferred by masking remain uncertain [122]. Additional areas for investigation include the effectiveness of immune globulin, prophylactic antimicrobials, and postexposure prophylaxis, as well as the potential cocoon effect of vaccinating close household contacts and caregivers to reduce transmission to vulnerable patients with cancer [12,123,124].

## 8. Conclusions

**Figure 1:**
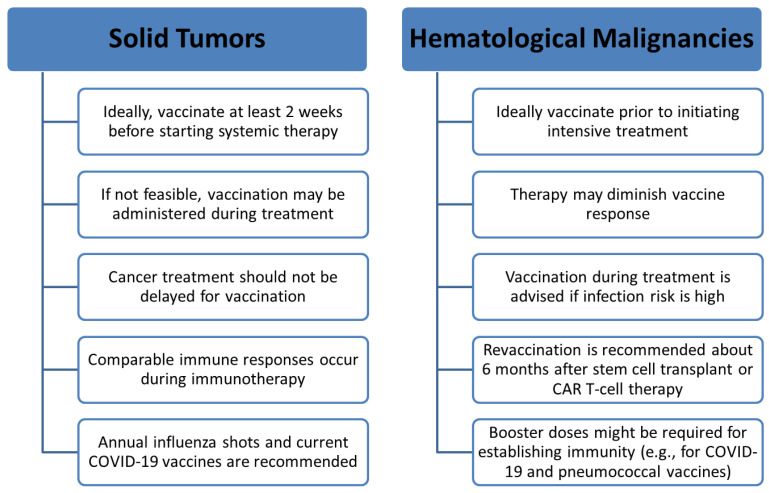
Recommended vaccination timing in adults with cancer.

Adults with cancer are at increased risk of severe respiratory infections, with substantial associated morbidity and mortality. Improving overall survival therefore requires not only effective anticancer therapy but also proactive prevention of respiratory infections, with vaccination forming a core component of holistic cancer care. Evidence from studies in both the general population and adults with cancer consistently demonstrates that the benefits of vaccination outweigh the risks, with adverse events occurring at rates comparable to those seen in individuals without cancer. The timing of vaccination is crucial—the earlier the better—and there is little justification for delaying vaccination even when immunogenicity may be attenuated (Figure 1). Vaccine dosing mirrors that recommended for the general population, with two key exceptions: live attenuated vaccines should be avoided because of the risk of replication and disease in immunocompromised patients, and selected populations may require booster doses to achieve adequate protection. Clinicians play a pivotal role in improving vaccine uptake—by systematically tracking vaccination status, issuing timely reminders, providing clear counseling on the risks of non-vaccination and the benefit–risk balance of vaccines, and ensuring that vaccination is delivered in the most accessible and convenient manner possible.

## Figures and Tables

**Table 1:** Vaccination against respiratory infections in adults with cancer *.

Pathogen	Vaccine Type	Target Population and Clinical Considerations	Timing Relative to Cancer Therapy
Influenza	Inactivated influenza vaccine. High dose or adjuvanted formulations where available	All adults with cancer. Reduced immunogenicity during intensive chemotherapy. Live attenuated vaccine contraindicated	Ideally before starting chemotherapy. May be given during treatment if needed. Annual administration before or during influenza season
COVID-19	mRNA vaccines. Protein subunit vaccines	All adults with cancer. Blunted responses in hematological malignancies. Serology is not routinely recommended to guide boosters	Preferably before systemic therapy. Can be given during active treatment. Avoid same day administration with intensive chemotherapy if possible
RSV	Recombinant protein and mRNA vaccines	Older adults with cancer. Consider younger adults with significant immunosuppression	Preferably before RSV season. Ideally prior to intensive cancer therapy
Pneumococcus	Conjugate vaccine followed by polysaccharide vaccine according to schedule. This series may be replaced by a high-valency conjugate vaccine (e.g., PCV20 or PCV21)	All adults with cancer. Polysaccharide vaccine alone has lower immunogenicity	Preferably before chemotherapy. Can be administered during treatment if not previously vaccinated
Pertussis	Tdap	Adults with cancer who have not received Tdap in adulthood or with uncertain history. Immunity wanes over time	Preferably before cancer therapy. Can be administered during treatment
Hib	Conjugate Hib vaccine	Adults who need stem cell transplantation for hematological malignancies	Preferably before chemotherapy. If not previously vaccinated, administer during or after treatment

Hib: *H. influenzae* type b; PCV: Pneumococcal conjugate vaccine; RSV: Respiratory syncytial virus; Tdap: Tdap: Tetanus toxoid, reduced diphtheria toxoid and acellular pertussis. * Consistent with various international guidelines [12,13,14,18].

**Table 2:** Initial vaccination and revaccination in adults with cancer *.

Pathogen	Initial Schedule	Patients Requiring Multiple Initial Doses	Revaccination Considerations
Influenza	Single dose	None at present	Annual vaccination. More frequent revaccination may be required in immunocompromised patients. Revaccinate about six months after stem cell transplantation regardless of prior history
COVID-19	Two-dose primary series followed by booster doses for immunocompromised patients	Patients receiving B-cell depleting therapy. Post-stem cell transplantation for hematological malignancies	Annual booster vaccination with variant-updated vaccine. More frequent booster may be required in immunocompromised patients. Serology is not routinely recommended to guide dosing. Revaccinate with full primary series about six months after stem cell transplantation regardless of prior history
RSV	Single dose. No current recommendation for multi-dose primary series	None at present	Currently single dose. Duration of protection and need for revaccination under evaluation. Revaccinate about six months after stem cell transplantation regardless of prior history
Pneumococcus	Conjugate vaccine followed by polysaccharide vaccine after recommended interval. This series may be replaced by a high valency conjugate vaccine (e.g., PCV20 or PCV21)	Post-stem cell transplantation for hematological malignancies	Revaccinate with a four-dose conjugate vaccine series about six months after stem cell transplantation regardless of prior history
Pertussis	Three-dose primary series if not previously vaccinated	Patients without prior adult Tdap or unknown vaccination history. Post-stem cell transplantation for hematological malignancies	Revaccinate every 10 years. Revaccinate with a three-dose series about six months after stem cell transplantation regardless of prior history
Hib	Three-dose primary series	Post-stem cell transplantation for hematological malignancies	Revaccinate with a three-dose series about six months after stem cell transplantation regardless of prior history

CLL: Chronic lymphocytic leukemia; Hib: *H. influenzae* type b; PCV: Pneumococcal conjugate vaccine; RSV: Respiratory syncytial virus; Tdap: Tetanus toxoid, reduced diphtheria toxoid, and acellular pertussis. * Consistent with various international guidelines [12,13,14,18].

## Data Availability

All data are presented in the article.
